# Metabolic Flux Redirection and Transcriptomic Reprogramming in the Albino Tea Cultivar ‘Yu-Jin-Xiang’ with an Emphasis on Catechin Production

**DOI:** 10.1038/srep45062

**Published:** 2017-03-23

**Authors:** Guo-Feng Liu, Zhuo-Xiao Han, Lin Feng, Li-Ping Gao, Ming-Jun Gao, Margaret Y. Gruber, Zhao-Liang Zhang, Tao Xia, Xiao-Chun Wan, Shu Wei

**Affiliations:** 1 State Key Laboratory of Tea Plant Biology and Utilization, Anhui Agricultural University, 130 Changjiang Ave W., Hefei, Anhui, 230036, China; 2 College of Life Sciences, Anhui Agricultural University, Hefei, Anhui, 230036, China; 3 Agriculture and Agri-Food Canada, Saskatoon Research Centre, Saskatoon, SK, S7N 0X2, Canada

## Abstract

In this study, shade-induced conversion from a young pale/yellow leaf phenotype to a green leaf phenotype was studied using metabolic and transcriptomic profiling and the albino cultivar ‘Yu-Jin-Xiang’ (‘YJX’) of *Camellia sinensis* for a better understanding of mechanisms underlying the phenotype shift and the altered catechin and theanine production. Shaded leaf greening resulted from an increase in leaf chlorophyll and carotenoid abundance and chloroplast development. A total of 1,196 differentially expressed genes (DEGs) were identified between the ‘YJX’ pale and shaded green leaves, and these DEGs affected ‘chloroplast organization’ and ‘response to high light’ besides many other biological processes and pathways. Metabolic flux redirection and transcriptomic reprogramming were found in flavonoid and carotenoid pathways of the ‘YJX’ pale leaves and shaded green leaves to different extents compared to the green cultivar ‘Shu-Cha-Zao’. Enhanced production of the antioxidant quercetin rather than catechin biosynthesis was correlated positively with the enhanced transcription of *FLAVONOL SYNTHASE* and *FLAVANONE/FLAVONOL HYDROXYLASES* leading to quercetin accumulation and negatively correlated to suppressed *LEUCOANTHOCYANIDIN REDUCTASE, ANTHOCYANIDIN REDUCTASE* and *SYNTHASE* leading to catechin biosynthesis. The altered levels of quercetin and catechins in ‘YJX’ will impact on its tea flavor and health benefits.

A group of albino *Camellia sinensis* cultivars, such as ‘Yu-Jin-Xiang’ (‘YJX’) and ‘Zhonghuang 2′, produce pale or yellow young leaves in early spring and turn green at later growing stages^1^,^2^,^3^,^4^. These cultivars are highly desired by the tea industry because young pale (virescent) tea leaves can be used to produce a high quality green tea with an enhanced savory (“umami”) taste and reduced stringency, while mature green leaves can maintain plant growth and regular productivity in successive years. Compared with the mature leaves of an albino cultivar and those of common green cultivars, young albino or yellow leaves usually contain enhanced levels of the non-protein amino acid theanine and reduced levels of catechins, a group of flavan-3-ols found in tea at high levels^1^,^2^,^4^. These metabolites are crucial tea flavor determinants. Catechins and their derivatives are mainly responsible for the bitter/astringent taste and deepen the color of tea infusions^5^. Theanine, a non-protein amino acid, contributes to the ‘umami’ taste and counteracts the astringent and bitter taste in tea infusions^6^.

Extensive studies on the biosynthesis of these specialized metabolites have revealed that catehcins are derived from the flavonoid pathway by the action of the biosynthetic genes dihydroflavonol 4-reductase (DFR)^7^, anthocyanidin reductase (ANR), leucoanthocyanidin reductase (LAR)^8^, and galloylated by the additional action of epicatechin:1-*O*-galloyl-β-*D*-glucose *O*-galloyltransferase (ECGT)^9^. Furthermore, the flavonoid pathway in plants is strictly regulated by transcription factors in a triprotein MYB-bHLH-WDR complex in response to different internal and external signals^10^. However, regulatory mechanisms underlying production of catechins in tea are still poorly understood. Biosynthesis of theanine is reportedly catalyzed by theanine synthase (TS) acting on two substrates (glutamate and ethylamine)^11^ to form theanine. Three additional enzymes, arginine decarboxylase (ADC), alanine decarboxylase (AIDA) or *S*-adenosylmethionine decarboxylase (SAMDC), might catalyze tea ethylamine production^12^,^13^, but this is not proven decisively. Moreover, *in vitro* enzyme assays showed bacterial γ-glutamyltranspeptidase (GGT) was able to synthesize theanine directly in an *in vitro* assay^14^. Hence, a thorough dissection of differentially expressed tea genes involved in these related pathways and an analysis of the correlation between gene expression and metabolite contents between pale and green leaves should enhance our understanding of the mechanisms underlying specialized tea metabolite biosynthesis and regulation in these two types of tea leaves.

Epigenetic regulation, important for gene transcription and phenotype reconstruction in response to developmental and environmental stimuli^15^,^16^, is likely involved (but not proven) in the leaf colour phenotype conversion observed in albino tea cultivars. In Arabidopsis, histone deacetylase HDA15 associated with PHYTOCHROME INTERACTING FACTOR3 regulates chlorophyll biosynthesis^17^. Moreover, biosynthesis of certain Arabidopsis leaf pigments (lutein and some other carotenoids) are epigenetically regulated by histone lysine methyltransferase SDG8^18^. In apple, methylation within the promoter of the *MdMYB10* gene, which regulates many flavonoid pathway genes upstream to anthocyanin and catechin biosynthesis, was supposedly responsible for the anthocyanin-deficient yellow-skin phenotype^19^. Several genes are also core elements controlling genome-wide epigenetic modification, including *DNAMETHYLTRANSFERASE1 (MET1*)^15^ and the methyl-CpG binding protein gene *MBD1*^20^. Thus, possible involvement of epigenetic regulation in tea leaf color alteration needs to learn.

Direct genetic mutations (as opposite to epigenetic regulation) of thousands of nuclear-encoded chloroplast proteins and more than a hundred plastidial genes could lead to aberrant chloroplasts and their malfunction^21^,^22^. To date, characterization of Arabidopsis chloroplast proteins using transposon- or T-DNA-tagged lines revealed that a pale or variegated leaf color can result from as many as 72 defective proteins^22^. Defective genes such as *E3 UBIQUITIN-PROTEIN LIGASE 1 (E3 RING1*) in the ubiquitination degradation pathway^23^ and *CLPR2, CLPR4*. and *CLPP3* in the caseinolytic protease (Clp) system can lead to pale green, yellow, and variegated leaf phenotypes^24^. Moreover, mutations in *1-DEOXY-D-XYLULOSE 5-PHOSPHATE SYNTHASE (DXS*) in the MEP pathway^25^,^26^ or in *GUN1*, a pentatricopeptide repeat-containing protein gene (PPR) functioning in the retrograde signaling pathway^27^,^28^, can also lead to albino phenotypes. Hence, determining global transcription changes with an emphasis on those genes related to pale leaf phenotypes of other species should help us to elucidate the mechanisms leading to the alteration in leaf color and specialized metabolite profiles found in tea.

In the current study, we compared transcriptomic and metabolic differences between ‘YJX’ pale green young leaves and their natural greening phenotype under shade treatment, with an emphasis on the changes in characteristic metabolites (catechin and theanine), defective chloroplasts and epigenetic regulation. Our data revealed defective chloroplast development, enhanced antioxidant biosynthesis (especially the redirection of catechins to the flavonol quercetin) and a role for SAMDC on theanine biosynthesis in the ‘YJX’ pale leaves.

## Results

### Leaf color variation and chloroplast ultrastructure observation

**Figure 1:**
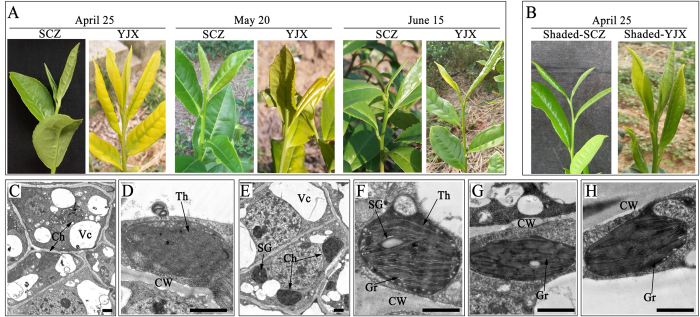
Alteration of leaf color and chloroplast ultrastructure of albino tea cultivar ‘YJX’ compared to the normal green cultivar ‘SCZ’. (**A**) Leaf color alteration over spring growing season of tea plants. (**B**) Four-day shaded ‘YJX’ pale leaves and ‘SCZ’ leaves. (**C**) and (**D**) chloroplasts in pale leaves of ‘YJX’ sampled on April 25^th^. (**E**) and (**F**) chloroplasts in the shaded green leaves of ‘YJX’ sampled on April 25^th^. (**G**) and (**H**) chloroplasts respectively in the non-shaded and shaded leaves of ‘SCZ’ sampled on April 25^th^. ‘YJX’, ‘Yu-Jin-Xiang’; ‘SCZ’, ‘Shu-Cha-Zao’; Ch, chloroplast; CW, cell wall; Th, thylakoid; Mi, mitochondria; Gr, grana; SG, starch granule; Vc, vacuole. Bars = 20 μm.

Visual and dynamic changes in leaf color of ‘YJX’ were observed over several growing seasons. In early spring (April in Hefei, China), newly generated young leaves of ‘YJX’ were pale green (PL) and then turned much paler (yellowed) and even contained scorched (dried out) edges as leaf development proceeded. By late spring (June), when shoot apical buds formed, the pale leaves recovered naturally to become green (Fig. 1A). A similar leaf color change occurred during the autumn growing season (data not shown), but all leaves of ‘YJX’ were green before the next year’s spring growing season started. Interestingly, pale or yellow leaves of ‘YJX’ recovered their green color under reduced light irradiation after shading for four days in spring (Fig. 1B), which were used for further analyses in this study. Transmission electronic microscopy (TEM) further revealed that non-shaded PL contained fewer and under-developed chloroplasts per transectioned cell (1.59/cell transection) than shade-induced green leaves (SL) (3.21/cell transection) of ‘YJX’ (Fig. 1C–F). SL chloroplasts maintained more visually clear thylakoid membrane systems with more intensively stained chloroplast components (grana stacks) and starch granules compared to PL chloroplasts, (1.20 and 0.61 granules per transectioned chloroplast, respectively) (Fig. 1D,F). These SL chloroplasts were generally comparable in chloroplast ultrastructure such as thylakoid membrane structure and inner component staining intensity to those in the leaves collected at the same developmental stage in ‘Shu-Cha-Zao’ (Fig. 1G,H), a common green leaf cultivar which is widely grown for regular green tea manufacturing in China. Our observations suggested that pale green leaves in ‘YJX’ had aberrant chloroplast development, but that reduced light intensity was able to recover (at least partially) normal leaf chloroplast development and leaf color.

### Metabolic alteration in leaf pigments and specialized tea metabolites

**Figure 2:**
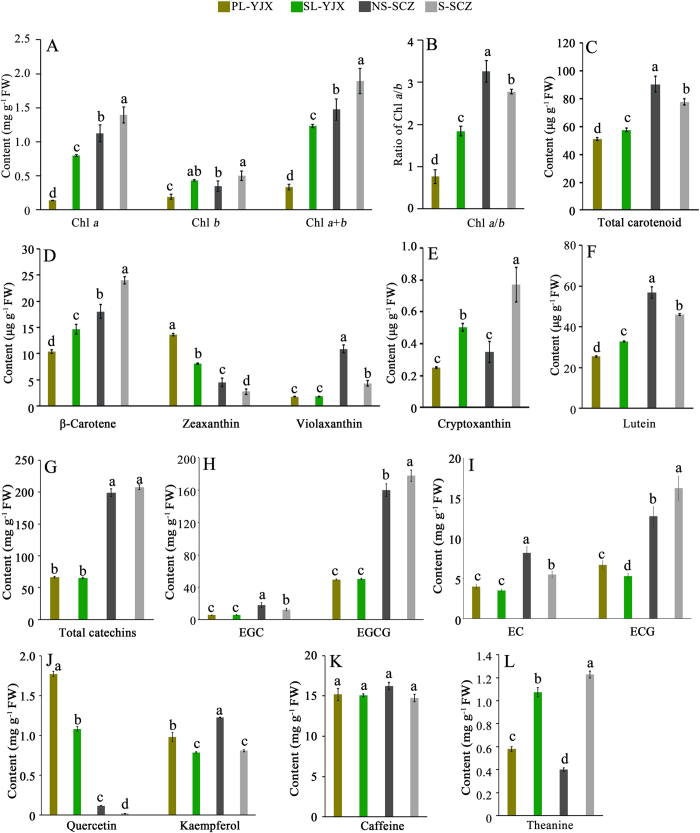
Alterations in leaf pigments and some specialized metabolites in the shaded and non-shaded leaves of ‘YJX’ and control ‘SCZ’. Duncan’s new multiple range test was performed. Columns labeled with the same letter had no significant difference among the four tested leaf measurements, *P* > 0.05. (**A**) Chlorophylls. (**B**) Ratio of chlorophyll *a/b*. (**C–F**) Abundances of total carotenoids, β-carotene, zeaxanthin, violaxanthin, cryptoxanthin, and lutein, respectively. (**G–I**) Abundances of total catechins, EGC, EGCG, EC, and ECG, respectively. (**J–L**) Abundances of quercetin, kaempferol, caffeine, and theanine, respectively. PL-YJX, pale leaves of ‘YJX’; SL-YJX, shaded green leaves of ‘YJX’; NS-SCZ, non-shaded leaves of ‘SCZ’; S-SCZ, shaded leaves of ‘SCZ’; Chl*a*, chlorophyll *a*; Chl*b*, chlorophyll *b*; EGCG, (−)-epigallocatechin gallate; EGC, (−)-epigallocatechin; EC, (−)-epicatechin; ECG, (−)-epicatechingallate. FW, fresh weight.

Consistent with the alterations in leaf color and chloroplast ultrastructure in the ‘YJX’ SL and PL, levels of chlorophyll-*a* (Chl*a*) and -*b* (Chl*b*), their sum (Chl*a* + *b*), and ratio (Chl*a/b*) in PL were dramatically lower than their corresponding values in SL of ‘YJX’ and even lower in leaves of ‘SCZ’ (*p* < 0.05) at the same developmental stage (Fig. 2A–C). In addition, in ‘YJX’ a significant reduction occurred (*P* < 0.05) in total carotenoids (11.1%), β-carotene (29.3%), cryptoxanthin (50.0%) and lutein (22.5%). However, a remarkable increase (*P* < 0.05) in zeaxanthin (69.1%) was found in PL compared to SL, while violaxanthin was at the same level in PL and SL (Fig. 2C–F). Moreover, the abundance of tested carotenoids in the shaded and non-shaded ‘YJX’ leaves were lower than their corresponding counterparts in ‘SCZ’ except for zeaxanthin, whose abundance was conversely higher in ‘YJX’ than in ‘SCZ’ (*P* < 0.05) (Fig. 2D).

Notably, significant changes were found in the abundance of specialized metabolites (flavonols, catechins, and theanine, but not caffeine) in ‘YJX’ compared to their counterparts in ‘SCZ’ (Fig. 2G–L). The levels of total catechins, four dominant catechines epigallocatechin 3-gallate (EGCG), epigallocatechin (EGC), epicatechin 3-gallate (ECG) and epicatechin (EC) in both shaded and non-shaded leaves of ‘YJX’ were all remarkably lower than those in ‘SCZ’ (*P* < 0.05) (Fig. 2G–I; Supplementary Fig. S1). A four-day shading treatment did not significantly affect the abundance of the tested catechins in ‘YJX’, except for ECG, which was reduced (*P* < 0.05). However, in ‘SCZ’, shading resulted in a significant increase in galloylated catechins EGCG and ECG (*P* < 0.05) (Supplementary Fig. S1) and a significant decrease in non-galloylated catechins EC and EGC (*P* < 0.05) (Fig. 2G–I). Interestingly, high level of quercetin, a product branched off from the metabolic flux towards catechin biosynthesis (Supplementary Fig. S1), was found in PL of ‘YJX’, followed by a significantly lower level in SL, and both were dramatically higher in the two types of ‘YJX’ leaves than in leaves of ‘SCZ’ (*P* < 0.05) (Fig. 2J). The abundance of kaempferol (Supplementary Fig. S1) in PL was also higher than that in SL (*P* < 0.05). Similarly, shading led to a significant reduction in kaempferol in ‘SCZ’ leaves (Fig. 2J). No difference was found in caffeine abundance among the different test leaves (Fig. 2K). However, a higher level of theanine was present in the non-shaded leaves of ‘YJX’ than in ‘SCZ’ (*P* < 0.05), whereas shade elevated theanine to a higher level in ‘SCZ’ leaves than in ‘YJX’ leaves (Fig. 2L).

### Transcriptomic alteration for various biological processes and pathways

**Figure 3:**
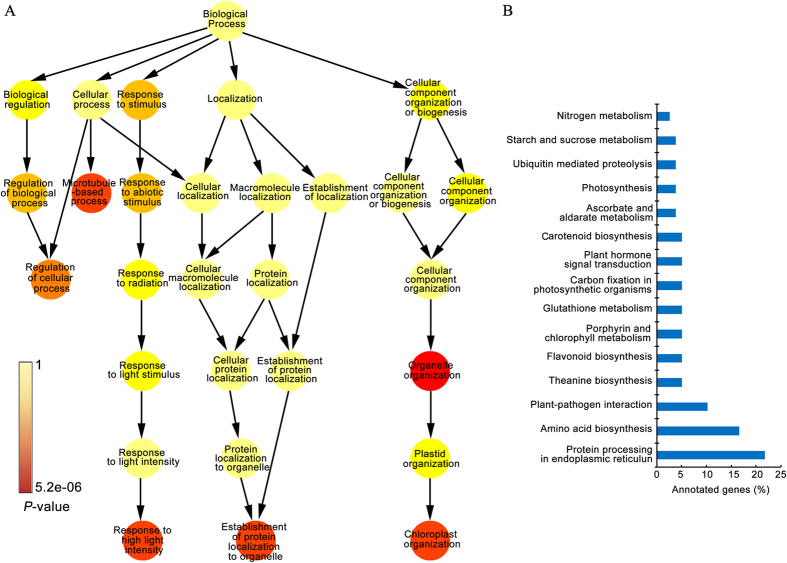
Overrepresented categories of the gene ontology biological processes and pathways by KEGG analysis based on the differentially expressed genes between the non-shaded pale and shaded green leaves in ‘YJX’. (**A**) Enriched gene ontology biological processes. (**B**) Affected metabolic pathways revealed by KEGG analysis. Theanine biosynthesis was constructed according to Kanehisa *et al*.^64^.

Transcriptomic analysis was conducted for ‘YJX’ non-shaded pale leaves and shaded green leaves based on a reference transcriptomic dataset established using “deep” sequencing of a mixed RNA from the tested leaf samples. In the reference dataset, a 18,1161 transcripts and 82,134 unigenes were assembled having N50 values 1,905 bp and 1,061 bp, respectively (Table 1; Supplementary Table S1; Supplementary Fig. S2). Further, a total of 37,753 unigenes were annotated using six publicly available databases (Table 1; Supplementary Table S2), while 1,196 differentially expressed genes (DEGs) were identified between the two types of leaf samples based on reads per kilobase per million (RPKM) and a fold change ratio limit of >2 (PL/SL) and *P-*value < 0.05^29^ (FDR Bonferroni-corrected). Gene Ontology (GO) analysis according to Alexa *et al*.^30^ revealed that the identified DEG dataset included significant differences in certain biological processes, molecular functions and cellular components (Supplementary Fig. S3), which were over-represented out of 355, 102, and 64, for the above three different categories respectively identified (data not shown). Enrichment analysis of the GO terms revealed that biological processes ‘chloroplast organization’, ‘response to high light’, and ‘establishment of protein localization to organelle’ were significantly affected (Fig. 3A) in addition to many other processes (Supplementary Fig. S3 and Table S5). KEGG analysis revealed the involvement of the DEGs in many biosynthetic pathways, particularly in protein processing, plant-pathogen interaction and amino acid biosynthesis, as well as theanine biosynthesis and flavonoid biosynthesis among other genes, each representing 5% of annotated genes (Fig. 3B).

Further analysis revealed that 44 DEGs were related to chloroplast activities, among which 13 were up-regulated and 31 were down-regulated in expression, and some gene products were localized in chloroplasts (Table 2). The 44 DEGs involved in diverse biological activities, and included the *LIGHT HARVESTING COMPLEX A-B BINDING PROTEIN* 7 (*LHCB7*) in photosynthesis, *ENDOGLUCANASE* 8 in carbohydrate metabolism, *TRANSLOCON OF THE INNER ENVELOPE OF CHLOROPLASTS* 32 (*TIC32*) in membrane transport, *THYMIDINE KINASE* in nucleotide metabolism, and the ATP-dependent *CLP PROTEASE PROTEOLYTIC SUBUNIT* 5 (*CLPP5*) involved in chloroplast protein catabolism. *SUCROSE SYNTHASE* 6, transporters and stress defensive genes were largely up-regulated in PL compared to SL, while others were down-regulated (Table 2). In addition, significant alteration was also found in transcript levels of five ubiquitous regulator-ubiquitin proteasome system genes (UPS), seven PPRs, two retrotransposon genes and one terpenoid pathway gene (Supplementary Table S3).

To determine potential epigenetic involvement in the alteration of leaf color and specialized metabolites, we identified 38 DEGs (out of all those identified) related to DNA methylation and histone methylation and acetylation in the ‘YJX’ transcriptome data. Interestingly, all 38 DEGs were lower in expression in PL compared to SL according to their RPKM ratios (Table 3). These included *DNA METHYLTRANSFERASE 1 (MET1*) (PL/SL = 0.29), *HISTONE-LYSINE N-METHYLTRANSFERASE ASHR* 3 (PL/SL = 0.21), *HISTONE-LYSINE N-METHYLTRANSFERASE ATXR* 6 (PL/SL = 0.24) and *METHYL-CPG-BINDING DOMAIN-CONTAINING PROTEIN* 9 (*MBD9*) (PL/SL = 0.43) (Table 3).

### DEG transcript level validation using quantitative real-time PCR

**Figure 4:**
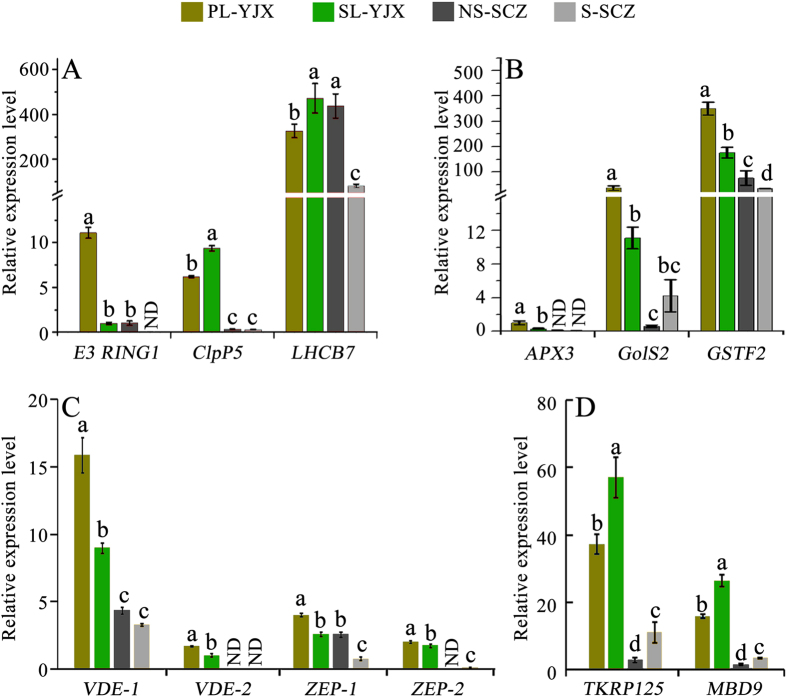
Validation of transcript levels of some of the DEGs using quantitative real-time PCR. (**A**) Transcript levels of DEGs related to chloroplast activities relative to *E3 RING1* in SL-YJX. *ClpP5, Clp protease proteolytic subunit 5*; *E3 RING1, E3 ubiquitin-protein ligase RING 1*; *LHCB7, Light harvesting chlorophyll a-b binding protein 7*. (**B**) and (**C**) Transcript levels of DEGs related to photo-protection relative to *APX3* in PL-YJX and *VDE-2* in SL-YJX. *APX3, ascorbate peroxidase3*; *GolS2, galactinol synthases 2*; *GSTF2, phi class of glutathione transferases 2*; *VDE, violaxanthin de-epoxidase*; *ZEP, zeaxanthin epoxidase*. (**D**) Transcript levels of DEGs related to epigenetics relative to *MBD9* in NS-SCZ. *TKRP125, 125* *kDa kinesin-related protein*; *MBD9, Methyl-CpG-binding domain-containing protein 9*. The columns labelled with the same letter for the same gene had no significant difference by Duncan’s new multiple range test, *P* > 0.05. ND, not detectable.

Quantitative real-time PCR (qPCR) analysis was applied for validation of transcriptomic data and also for differentiation of gene expression under shade and non-shade conditions used to test the two cultivars ‘YJX’ and ‘SCZ’. QPCR results confirmed that ‘YJX’ non-shaded pale leaves had a significantly higher transcript level of the *E3 RING1* gene, but significantly lower levels of *ClpP5* and *LHCB7* (Fig. 4A) compared to shaded green YJX’ leaves. Similarly, the expression of *E3 RING1* in ‘SCZ’ was significantly lower in non-shaded leaves compared to shaded leaves. *ClpP5* had very low expression in ‘SCZ’ in both non-shaded and shaded leaves compared to their counterparts in ‘YJX’ (Fig. 4A). Moreover, *LHCB7* expression was significantly reduced due to shading in ‘SCZ’, which was opposite to what we found in ‘YJX’ (Fig. 4A).

The expression levels of *ASCORBATE PEROXIDASE 3 (APX3*), *GALACTINOL SYNTHASE 2 (GolS2*), *GLUTATHIONE TRANSFERASE 2 (GSTF2*), *VIOLAXANTHIN DE-EPOXIDASE (VDE*) and *ZEAXANTHIN EPOXIDASE (ZEP*), related to the biological process ‘response to high light’ in GO analysis were also examined by qPCR. Transcript levels of all these unigenes were higher in PL than in SL in ‘YJX’ (*P* < 0.05) (Fig. 4B,C). However, the expression of these genes (*P* < 0.05) in ‘SCZ’ shaded and non-shaded leaves were not significantly changed, except for *GSTF2* which had a lower expression level in shaded leaves than in shaded ones (Fig. 4B). *VDE* and *ZEP* had higher expression in PL than in SL (Fig. 4C), a finding which correlated well with the difference in violaxanthin and zeaxanthin (Fig. 2D–F). Notably, *VDE-2* and *ZEP-2* were expressed at very low levels in ‘SCZ’ in both shaded and non-shaded leaves (Fig. 4C). These data suggested that the photo-protection system had a higher expression level in PL of ‘YJX’ than in SL and ‘SCZ’. Moreover, the transcript levels of the *125 KDA KINESIN-RELATED PROTEIN* (TKRP125) and *MBD9* genes were significantly lower in the non-shaded leaves than in the shaded leaves in both cultivars (*P* < 0.05). As well, all qPCR data were highly consistent with the transcriptomic results.

### Differentially expressed genes in the flavonoid pathway

Flavonoid-derived phenolic compounds, especially the dihydroxyflavonol quercetin, are specialized compounds not only functioning as antioxidants in photo-protection to quench reactive oxygen species^31^, but also determining tea flavor and having health. Unigenes annotated as *CHALCONE SYNTHASE (CHS) -2* and *-3* were found within our transcriptomic data out of the three *CHS* isoforms previously reported in tea^32^,^33^. Sequences common to both *CHS-2* and *-3* were used for qPCR primer design (Supplementary Table S3) to quantify these two isoforms together. A full length *FLAVONOL SYNTHASE (FLS*) cDNA was found to be identical to the posted sequence (GenBank EF205150.1). In addition to the functionally characterized *LAR1* in tea (Genbank GU992401)^8^, *LAR2*, which was previously reported to have a lower expression than *LAR1*^33^, was found. For *FLAVANONE 3*′*-HYDROXYLASE (F3*′*H*), *CHALCONE ISOMERASE (CHI*), and *ANTHOCYANIDIN SYNTHASE (ANS*), quantification of their transcripts was performed only using annotated unigenes with the longest sequences, since these three genes had not been previously characterized so far and the longer sequences would be functionally more reliable than short assembled sequences present in the transcriptome data.

**Figure 5:**
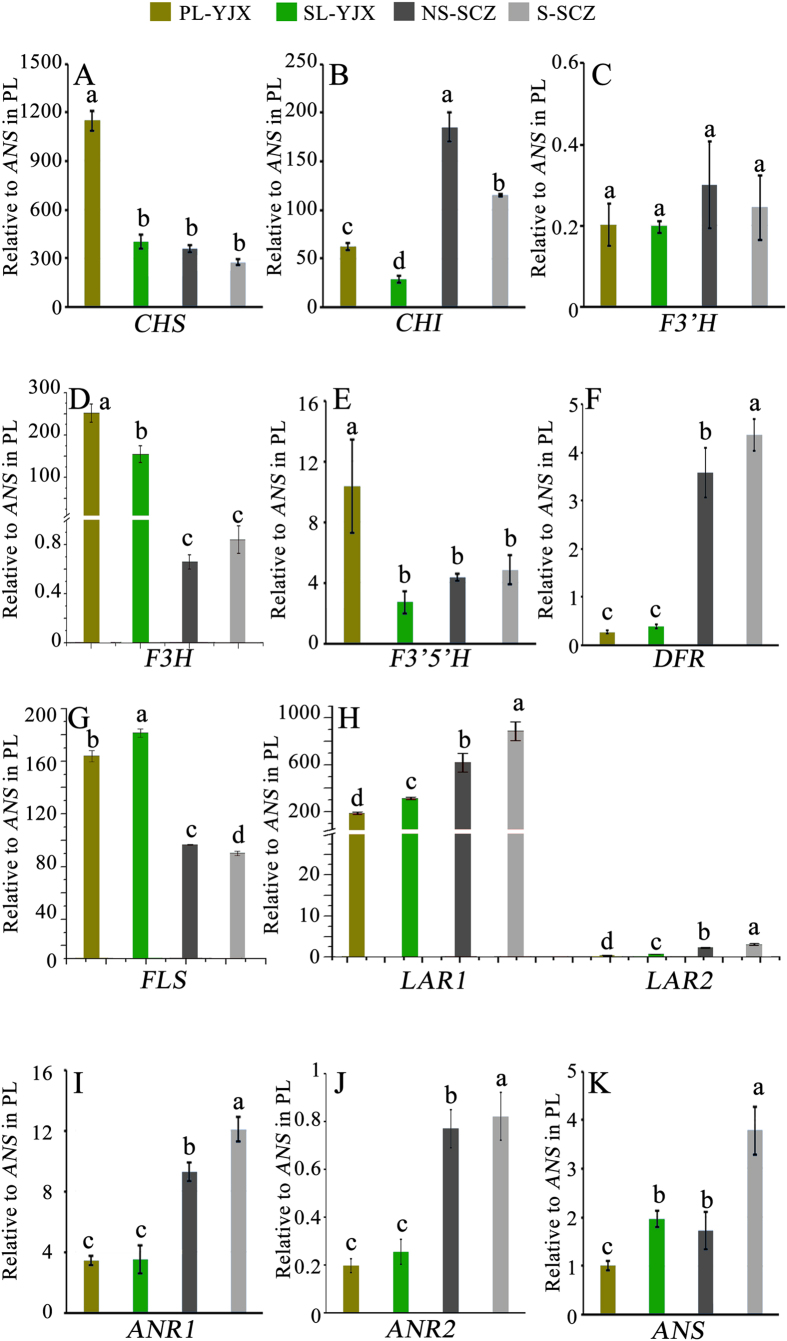
Transcript levels of some genes in flavonoid pathway in the ‘YJX’ and ‘SCZ’ shaded and non-shaded leaves relative to *ANS* level in the ‘YJX’ pale leaves. Duncan’s new multiple range test was performed and the levels labeled with the same letter for the same gene exhibited no significant difference among the tested samples, *P* > 0.05. *CHS, chalcone synthase*; *CHI, chalcone isomerase*; *F3H, flavanone 3-hydroxylase*; *F3′H, flavonoid 3′-hydroxylase*; *F3′5′H, flavonoid 3′,5′-hydroxylase*; *DFR, dihydroflavonol 4-reductase*; *FLS, flavonol synthase*; *LAR, leucoanthocyanidin reductase*; *ANR, anthocyanidin reductase*; *ANS, anthocyanidin synthase*.

The transcript levels of *CHS, CHI, F3H,* and *FLAVONOID 3′,5′-HYDROXYLASE (F3*′*5*′*H*), all functioning in the upstream pathway, were significantly higher (*P* < 0.05) in PL than in SL of ‘YJX’, while *DIHYDROFLAVONOL 4-REDUCTASE (DFR*) maintained the same level in both leaf types and *FLAVONOL SYNTHASE (FLS*) was expressed at a lower level in PL than in SL of ‘YJX’ (Fig. 5). In ‘SCZ’, the expression of several of these genes was almost at the same level under shading and non-shading conditions, except for *CHI, DFR*, and *FLS (P* < 0.05) (Fig. 5). Significantly higher expression of *CHI* and *FLS* and lower expression of *DFR* were found in the non-shaded leaves compared to the shaded leaves in ‘SCZ’ (Fig. 5F,G). Moreover, high expression of *FLS* and low expression of *DFR* were noted in ‘YJX’ compared to ‘SCZ’. However, the expression of downstream pathway genes *LAR1* and -*2, ANS* and *ANTHOCYANIDIN REDUCTASE (ANR) -1* and -*2* were lower (*P* < 0.05) in PL than in SL in ‘YJX’, and their expression showed the same pattern in ‘SCZ’ (Fig. 5H–J). These results suggested that transcription changes in flavonoid pathway genes favored enhanced biosynthesis of quercetin rather than catechins in ‘YJX’.

### Differentially expressed genes in the theanine biosynthetic pathway

Theanine was enriched in PL of ‘YJX’ compared with common green tea cultivars^1^,^4^. For a better understanding of theanine production in ‘YJX’ and ‘SCZ’ under shade conditions, expression of genes supposedly related to theanine biosynthesis was examined except for *ARGININE DECARBOXYLASE (ADC*) and *ALANINE DECARBOXYLASE (AIDA*) since no annotated unigenes were found in our transcriptome data. For transcript quantification, all unigenes annotated as *ALANINE AMINOTRANSFERASE (ALT*) and *GGT* were quantified using qPCR. *GLUTAMATE DEHYDROGENASE (GDH*) -*1* and *SAMDC* were also chosen for qPCR since our transcriptomic data indicated that they were differentially expressed between PL and SL (data not shown). For other related genes, the unigenes with the longest sequences were quantified.

**Figure 6:**
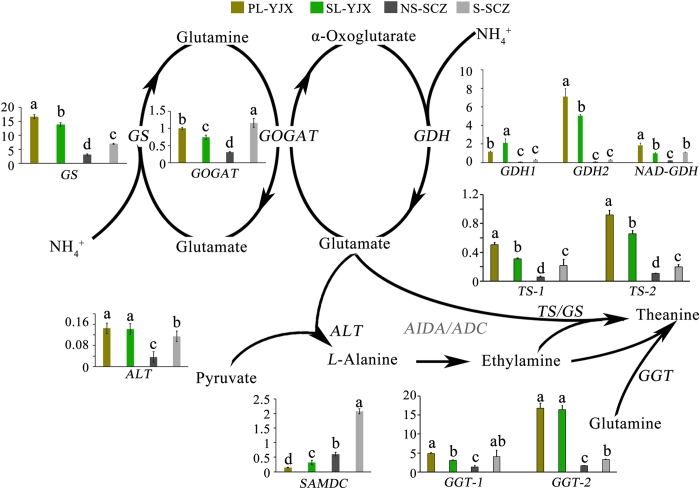
Transcript levels of the genes for tea theanine biosynthesis in the shaded and non-shaded leaves of ‘YJX’ compared to the counterparts in ‘SCZ’. Duncan’s new multiple range test was performed and the levels labeled with the same letter for the same gene exhibited no significant difference among the tested samples, *P* > 0.05. Transcript level of *GOGAT* gene was set as 1. *GS, glutamine synthetase*; *GOGAT, glutamate synthase; GDH, glutamate dehydrogenase*; *NAD-GDH: NAD-dependent glutamate dehydrogenase; TS, theanine synthetase*; *ALT, alanine aminotransferase*; *SAMDC: S-adenosylmethionine decarboxylase; AIDA, alanine decarboxylase*; *ADC, arginine decaboxylase*; *GGT, γ-glutamyltranspeptidase*.

The expression levels of *GLUTAMINE SYNTHETASE (GS*), *GLUTAMATE SYNTHASE (GOGAT*), *GLUTAMATE DEHYDROGENASE (GDH-2*), and *NAD-GLUTAMATE DEHYDROGENASE (NAD-GDH*), all related to the bioconversion between glutamate and glutamine, were enhanced in the non-shaded PL compared with the shaded SL of ‘YJX’ (Fig. 6). The expression of *ALANINE AMINOTRANSFERASE (ALT*), responsible for the biosynthesis of the precursor L-alanine, was unchanged between PL and SL. In contrast, the expression of *SAMDC*, responsible for the biosynthesis of the immediate precursor ethylamine, was lower in PL than in SL. *TS*-*1, TS*-2, and *GGT-1, GGT-2*, catalyzing theanine production, were expressed higher in PL than in SL of ‘YJX’. Interestingly, transcription levels of all the tested genes in ‘SCZ’ were higher in shaded leaves than in non-shaded ones and consistent with the contents of theanine in ‘SCZ’.

## Discussion

In this study, phenotypic alteration, transcriptomic reprogramming and metabolic flux redirection were studied using the conversion of a young pale/yellow leaf phenotype into a green leaf phenotype in the albino tea (*Camellia sinensis*) cultivar ‘YJX’ under shaded conditions. By comparing this conversion with changes that occurred in leaves of the common green tea cultivar ‘SCZ’ under the same shade conditions, dynamic differences in ‘YJX’ were elucidated.

Phenotypic alterations in leaf color and specialized metabolite abundance occurred with leaf development and light intensity in ‘YJX’. Natural high light shining upon pale leaves in spring led to a more extreme pale phenotype, whereas reduced light intensity through shading resulted in the rapid greening of leaf color of ‘YJX’. Similar light dependent leaf phenotypic changes have been reported previously in maize and Arabidopsis^34^,^35^. Our data revealed that the non-shaded pale leaves of ‘YJX’ contained aberrant chloroplasts with undeveloped thylakoid membranes and significantly reduced chlorophylls and total carotenoids, both of which play crucial roles in photosystem assembly and, light-harvesting and the latter in photo-protection in chloroplasts^36^,^37^, were found in PL compared to SL of ‘YJX’. Moreover, a dramatic reduction for four catechins and an increase for zeaxanthin occurred in ‘YJX’ compared to ‘SCZ’, as reported previously in some other albino tea cultivars^1^. In addition, a high level of quercetin was found in PL, followed by that in SL of ‘YJX’, and both were tremendously higher than their counterparts in ‘SCZ’ leaves. This is highly consistent with the finding that plants undergoing severe stress conditions (such as high light stress) preferentially accumulate more effective antioxidants such as the dihydroxy B-ring-substituted flavonoid quercetin^36^,^38^ and the carotenoid zeaxanthin (a crucial component of the violaxanthin cycle in plant photo-protection)^36^,^37^,^39^. These results indicated that metabolic flux redirection towards zeaxanthin and quercetin enhancement occurred in PL and SL of ‘YJX’ to varying extents compared to ‘SCZ’.

Interestingly, catechin abundance between non-shaded PL and shaded SL of ‘YJX’ was not significantly different, while in shaded leaves of ‘SCZ’ the galloylated catechins (EGCG and ECG) were higher and non-galloylated catechins (EGC and EC) lower than in non-shaded leaves (*P* < 0.05). This finding in the green cultivar ‘SCZ’ was consistent with previous reports on the changes in the abundance of galloylated and non-galloylated catechins in shaded and non-shaded tea leaves^40^,^41^. However, the data for albino ‘YJX’ differed from a previous report^42^ in which a significant reduction occurred in catechin levels after 3 weeks of shading compared to non-shaded green tea cultivar^42^. This difference could result from a change in metabolic flow in albino tea leaves and from differently applied shading periods compared with our study^43^. Significant difference in the abundance of carotenoids, chlorophylls, quercetin, and theanine were also noted between PL and SL of ‘YJX’ and compared to their counterparts in ‘SCZ’ leaves. These data suggest that regulation of catechins differ from that of the other metabolites we studied. Metabolic flux redirection was also reported for theanine enhancement under shade condition as reported for some other albino tea cultivars^1^,^4^. Due to the multiple health benefits and flavor contributions of quercetin^5^ and theanine^6^, it would be interesting to find out the possible changes in tea flavor and health functions of the albino ‘YJX’.

Transcriptome reprogramming was observed in the PL of ‘YJX’ based on establishment of a deep sequencing reference transcriptome since tea genome information is still unavailable. A total of 1,196 DEGs were identified between the ‘YJX’ PL and SL, which compared favorably with DEG profiles found in Arabidopsis under high light stress^44^. For validation of the identified DEGs, qPCR was performed on a small subset of DEGs, including *E3 RING1, ClpP5, LHCB7* related to aberrant chloroplasts, *APX3, GolS2, GSTF2, VDE* and *ZEP* related to photo-protection, and *TKRP125* and *MBD9* related to epigenetic regulation. GO enrichment analysis revealed that a wide range of biological processes and metabolic pathways were likely affected by these DEGs, including ‘chloroplast organization’, ‘responses to high light intensity’, ‘flavonoid biosynthesis’ and ‘amino acid biosynthesis’. However, light sensor genes^45^, including *PHYTOCHROME A/B/E, CRYPTOCHROME 1, PHOTOTROPIN 1/2* and *UV RESISTENCE LOCUS 8*, were found not significantly changed in shaded and non-shaded ‘YJX’ leaves. Moreover, downstream genes in the light transduction pathways proposed by Chory^45^ were neither affected by the shade treatment, nor found in our transcriptomic data. It is interesting to learn the roles of light transduction pathways in the observed transcriptomic and metabolic changes in this study. Consistent with the aberrant chloroplasts we observed, a total of 44 DEGs related to chloroplast activities were found in PL. Interestingly, an extraordinarily high expression level (>18 fold) of *ClpP5*, a component of the Clp proteasomal complex in the chloroplast, was found in in ‘YJX’ (SL > PL) compared to the control cultivar ‘SCZ’. Abnormal transcription of *ClpP5* in maize^34^ and *Arabidopsis*^35^ also results in a virescent leaf phenotype, which can be restored by reduced light intensity. Further investigations are required to find out whether *ClpP5* or other subunits of the Clp complex is responsible for the leaf color phenotypic alteration in ‘YJX’.

Chlorophyll deficiency in albino plants generally results in a host of responses to high light stress^46^ not only by enhancing antioxidant metabolites (including ascorbate, glutathione^47^,^48^, zeaxanthin^36^,^37^,^39^, lutein^36^, and quercetin^36^,^38^) but also reprogramming to activate transcriptional responses to high light intensity. Transcriptomic reprogramming also occurred in ‘YJX’ to the genes related to epigenetic regulation, as expected, since the observed leaf phenotypic conversion in ‘YJX’ was activated by plant development and shading, both of which are processes governed by epigenetic modification^15^. Our data suggested that the 36 DEGs involved in DNA and histone methylation, including H3K27 trimethylation, likely regulate light-induced gene transcription as seen in many other plant species such as Arabidopsis and tobacco^49^,^50^. Thus lower expression of these methylation genes negatively correlated with the higher expression of most tested genes in non-shaded leaves compared to shaded ones both in ‘YJX’ and ‘SCZ’^15^. More detailed investigations are needed for determining the role of specific epigenetic genes on the ‘YJX’ pale phenotype.

Since quercetin was enhanced and catechins decreased in ‘YJX’, flavonoid pathway gene expression was also examined with a focus on metabolic flux redirection in ‘YJX’. Catechins are highly abundant in tea leaves, and their biosynthesis and accumulation have been extensively studied in this species^7^,^8^,^9^. In tea plants, LAR and ANR^8^, F3′5′H^51^, and ECGT^9^ are key downstream enzymes catalyzing the biosynthesis of catechins. Nevertheless, functional characterization of genes for catechin biosynthesis is still limited in tea plants^8^,^51^. In this study, qPCR results showed that expression of many upstream flavonoid genes such as *CHS, CHI*, and *F3H*, were significantly enhanced in PL compared to SL of ‘YJX’, suggesting the possibility of an enhanced metabolic flux within this part of flavonoid pathway. Higher expression levels of *F3H* and *F3*′*5*′*H* in PL, genes which are responsible for dihydroflavonol and flavonol quercetin biosynthesis^52^, were also consistent with an enhanced level of quercetin in PL. Lower levels of *ANR1* and *-2, ANS*, and *LAR1* and *-2* in ‘YJX’ than in ‘SCZ’ also correlated with decreased levels of different catechins in ‘YJX’. Furthermore, high expression of *FLS* and low expression of *DFR* in ‘YJX’ compared to ‘SCZ’ was consistent with the difference in flavonol and flavan-3-ols between the two cultivars. This consistency is further supported by the functional characterization of the two genes^7^,^31^,^53^ and *FLS* expression is also correlated negatively to catechin abundance in tobacco^50^ and tea^3^, but positively correlated with quercetin^53^. Our data suggested that these genes could be ‘turning points’ for the metabolic flux redirection in ‘YJX’.

The enhanced level of theanine in albino leaves or by shade observed in this study and reported earlier^1^,^4^,^54^,^55^ suggested potential transcription changes for genes related to theanine biosynthesis. Theanine is reportedly synthesized by theanine synthetase from glutamic acid and ethylamine^56^, which is derived from L-alanine in tea plants^11^,^57^ by the action of AIDA, ADC or SAMDC^12^,^13^. However, only *SAMDC* out of these three decarboxylases was mapped in our tea transcriptome data^14^,^15^. This higher expression of *SAMDC* was consistent with the variation of theanine abundance in shaded and non-shaded leaves of the two cultivars. Additionally, all theanine genes tested by qPCR were also expressed at a higher level in shaded ‘SCZ’ leaves than in non-shaded ones, consistent with the change in theanine level between the two types of ‘SCZ’ leaves but not in ‘YJX’. Hence, the underlying biological mechanisms underlying theanine biosynthesis in ‘YJX’ remain unclear.

In summary, alterations in leaf color, leaf pigments, flavonoids, and theanine as well as global transcription specifically related to flavonoid and theanine pathways clearly revealed that metabolic flux redirection and transcriptomic reprogramming occurred in the pale leaves of ‘YJX’ compared with shaded leaves of this cultivar and the green cultivar ‘SCZ’. Consistency between dramatically altered abundance of multiple metabolites and expression of multiple corresponding genes in flavonoid pathway in ‘YJX’ suggested the action of a regulatory mechanism in ‘YJX’ underlying a concerted expression regulation of many genes upon high light stress. The identified differentially expressed genes related to chloroplast activities and epigenetic regulation may lead to a new horizon to elucidate mechanisms underlying over the ‘YJX’ leaf phenotypic conversion. Our study also has a great potential for tea flavour and health benefit improvement.

## Methods

### Plant material and shading treatment

Six-year old plants of *Camellia sinensis* cv. ‘Yu-Jin-Xiang’ (‘YJX’) and ‘Shu-Cha-Zao’ (‘SCZ’) were grown in the tea farm of Anhui Agricultural University located at 31°55′42.8″N, 117°12′09.1″E; Hefei City, Anhui, China with identical cultivation management. Shade treatment was started for young pale leaves of ‘YJX’ and ‘SCZ’ leaves at the same developmental stage on April 22, 2015 for four consecutive days using two layers of black shading nets, resulting in light intensity reduced to 18 mol m^−2^ s^−1^ (12.5% of natural sunlight intensity). After four days of treatment, shaded and non-shaded leaves of ‘YJX’ and ‘SCZ’ were collected for chemical profiling, electronic microscope observations, or in some cases transcriptomic sequencing. In detail, TEM, chemical and qPCR analyses were performed using the four types of leaves to give direct comparisons. For transcriptomic data analysis, non-shaded pale and shaded re-greening leaves in ‘YJX’ were used since the changes in ‘YJX’ over the leaf color conversion were focused and leaf transcript analysis of normal green cultivars under shade and non-shade conditions had been reported before^42^,^58^.

### RNA Isolation, RNA-Seq library construction and illumina sequencing

Total RNA was extracted from tea leaves by using the RNAprep pure Plant Kit (TianGen Biotech., Ltd, Beijing, China). RNA quality and quantity were determined using both agarose gels and a Nanodrop 2000 spectrophotometer (Thermo Fisher Scientific, Wilmington, DE, USA). RNA samples with A_260/280_ ratio between 1.8 to 2.0, A_260/230_ ratio between 2.0 to 2.2 and RIN (RNA integrity number)^59^ more than 8.0, were used for transcriptomic sequencing. Purified RNA samples (one mixture of young, old and shading leaves and three biological replicates each for PL and SL) were sent to Biomarker Technologies Corporation (Beijing, China) for cDNA library construction and sequencing. For each sample, 10 ng RNA was used for cDNA synthesis with oligo (dT) for mRNA enrichment. Enriched mRNA was fragmented and amplified using an Ovation RNA-Seq System V2 kit (Nugen Technologies, San Carlos, USA) following the manufacturer’s protocols. The cDNA was purified and 1 μg amplified cDNA for each sample was used to generate multiplexed RNA-Seq libraries (mean size about 360 bp) and sequenced using the Illumina HiSeq^TM^ 2000 platform (Illumina, Inc., Shanghai, China).

### Functional annotation of the unigenes

The unigene sequences of the mixed tea leaf sample aligned using BLASTX against the COG, GO, KEGG, Swissprot, TrEMBL and NR databases (E-value < = 1E-5) to retrieve functional annotations based on sequence similarity. Unigenes that could not be aligned to any of these databases were analyzed by the ESTScan software^60^. Gene ontology (GO) analysis was conducted using the TopGO package^30^. The GO terms with p-values <0.05 were considered significantly enriched. The biological processes of enriched GO terms were then visualized using TopGO^30^.

### Differentially expressed genes functional enrichment

All usable reads were normalized into RPKM values (reads per kb per million reads)^61^. Differential expression of unigenes between two RNAseq samples were calculated based on “base mean” value obtained from the DESeq package. Only unigenes with an PL/SL RPKM ratio >2 and a false discovery rate (FDR) multiple test score of *P* < 0.05^29^, were considered to be differentially expressed unigenes.

### Chloroplast ultrastructure observation and chlorophyll abundance measurement

For ultrastructural observation, fresh leaves were excised and infiltrated with 4% glutaraldehyde solution using a syringe and then soaked in the solution according to Li *et al*.^4^. Glutaraldehyde-infiltrated tea leaves were cut into 2 × 2 mm pieces and further sectioned using a TCS CM1900 freezing microtome (Leica, Germany). The ultrathin section was double lead stained according to Daddow *et al*.^62^ and then observed using a HT-7700 transmission electron microscope (TEM) (Hitachi, Japan).

Fresh leaves (0.1 g, accurate to 0.001 g) were used for chlorophyll abundance quantification according to Feng *et al*.^1^. The excised fresh leaves were cut into small pieces. Chlorophyll was extracted overnight using 10 mL (5% acetone: 95% ethanol, v/v) until the leaves became completely white, then the extract was measured using an ultraviolet spectrophotometer (U-5100, Hitachi, Japan) at A_645_ and A_663_. The chlorophyll contents were calculated using the following formula according to Feng *et al*.^1^:


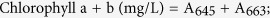



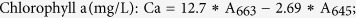



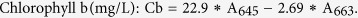


All experiments were replicated using three independent harvests and light treatments using 20 leaves and 3 tea plants per replicate, and illustrated as ±SD. Duncan’s new multiple range test was performed for significance analysis.

### Quercetin and kaempferol, catechin and carotenoid content

Extraction and quantification of catechins and carotenoids were carried out as previously reported^1^. The extraction of quercetin and kaempferol prior to hydrolysis was carried out following the protocol of Xiong *et al*.^3^: tea leaves (ca. 1 g dry weight) were mixed with 40 ml of 60% ethanol and 5 ml HCl (6 M). After refluxing at 95 °C for 2 h, the hydrolyzed solutions were filtered through filter paper, then diluted to 50 ml each using 60% ethanol. Abundances of flavonols, catechins and carotenoid were measured according to previous reports^1^,^3^, using HPLC with a C18 Column (4.6 × 250 mm; Phenomenex, Shanghai, China). The compounds were quantified using the standard curves. All compounds were measured using three replicates and shown as means ± SD. Statistical analysis was performed using Duncan’s new multiple range test.

### Quantitative real-time PCR

Q-PCR expression assays were performed on a CFX96 platform (Bio-Rad, www.bio-rad.com/), using specific primers (listed in Table S4), and the Top Green qPCR SuperMix (TransGen Biotech, Beijing, China) according to the manufacturer’s instructions. PCR reaction efficiencies for all test genes were over 90% and their transcript levels were calculated using the 2^−ΔΔCt^ method^63^. All experiments were done using three replicates and shown as means ± SD. Duncan’s new multiple range test was performed for significance analysis.

## Additional Information

**How to cite this article:** Liu, G.-F. *et al*. Metabolic Flux Redirection and Transcriptomic Reprogramming in the Albino Tea Cultivar ‘Yu-Jin-Xiang’ with an Emphasis on Catechin Production. *Sci. Rep.* **7**, 45062; doi: 10.1038/srep45062 (2017).

**Publisher's note:** Springer Nature remains neutral with regard to jurisdictional claims in published maps and institutional affiliations.

## Supplementary Material

Supplementary Information

## Figures and Tables

**Table 1:** Transcriptomic data assembly and annotation.

Assembly							
	Total number		Total length/bp		N50/bp		
Transcripts	181161		198943185		1905		
Unigenes	82134		52097841		1061		
**Annotation**							
**Database**	**COG**	**GO**	**KEGG**	**Swissprot**	**TrEMBL**	**NR**	**All**
Annotated number	8459	23663	5864	20807	33862	33780	37753

**Table 2:** Differentially expressed genes related to chloroplast activity.

Unigene	Name	GO terms	PL/SL
**Only located in chloroplast**			
c51594.graph_c3	Alpha-glucan water dikinase 1, chloroplastic	Chloroplast envelope (GO:0009941)	2.67
c35497.graph_c1	Partial cpsHSP gene for chloroplast small heat shock protein	Chloroplast (GO:0009507)	2.51
c24665.graph_c0	beta-1,3-galactosyltransferase 19	Chloroplast (GO:0009507)	2.01
c45824.graph_c1	Microtubule-associated protein TORTIFOLIA1	Chloroplast (GO:0009507)	0.49
c43126.graph_c2	Uncharacterized protein	Chloroplast (GO:0009507)	0.45
c29549.graph_c1	Translocon of the inner envelope of chloroplasts TIC 32, chloroplastic	Chloroplast inner membrane (GO:0009706)	0.43
c37006.graph_c0	D-3-phosphoglycerate dehydrogenase, chloroplastic (Precursor)	Chloroplast (GO:0009507)	0.41
c22156.graph_c0	Chlorophyll a-b binding protein 7, chloroplastic	Chloroplast thylakoid membrane (GO:0009535)	0.39
c54551.graph_c0	Thymidine kinase	Chloroplast (GO:0009507)	0.36
c48922.graph_c0	Glucose-6-phosphate 1-dehydrogenase 2, chloroplastic (Precursor)	Chloroplast stroma (GO:0009570)	0.35
c61815.graph_c0	ATP-dependent Clp protease proteolytic subunit 5, chloroplastic	Chloroplast envelope (GO:0009941)	0.31
c58182.graph_c0	Elongation factor G, chloroplastic (Precursor)	Chloroplast stroma (GO:0009570)	0.28
c49731.graph_c2	Probable anion transporter 3, chloroplastic (Precursor)	Chloroplast (GO:0009507)	0.28
**Not only or not located in chloroplast**			
c41177.graph_c3	Sucrose synthase 6	Chloroplast (GO:0009507)	7.75
c26017.graph_c0	L-ascorbate peroxidase 3, peroxisomal-like	Chloroplast envelope (GO:0009941)	6.76
c57671.graph_c0	Mitochondrial adenine nucleotide transporter BTL3 GN = At5g64970	Plastid (GO:0009536)	2.92
c31428.graph_c5	Phospholipase D alpha	Chloroplast envelope (GO:0009941)	2.72
c17671.graph_c0	Potassium transporter 4	Chloroplast (GO:0009507)	2.51
c20478.graph_c0	Calcium-binding mitochondrial carrier protein SCaMC-1-like	Plastid (GO:0009536)	2.34
c47097.graph_c1	Predicted protein [Populus trichocarpa]	Chloroplast (GO:0009507)	2.24
c30056.graph_c0	Aconitase, putative (EC:4.2.1.3)	Chloroplast (GO:0009507)	2.17
c38078.graph_c1	Molybdate transporter 1	Chloroplast (GO:0009507)	2.13
c38410.graph_c0	L-ascorbate peroxidase 3, peroxisomal-like	Chloroplast envelope (GO:0009941)	2.09
c14566.graph_c0	Endoglucanase 8 (Precursor)	Chloroplast (GO:0009507)	0.49
c46977.graph_c1	Inactive leucine-rich repeat receptor-like protein kinase At1g66830	Chloroplast stroma (GO:0009570)	0.47
c42606.graph_c1	Dolichyl-diphosphooligosaccharide-protein glycosyltransferase subunit STT3A	Chloroplast (GO:0009507)	0.46
c69625.graph_c0	Serine/threonine-protein kinase ATR	Plastid (GO:0009536)	0.46
c21206.graph_c1	60S ribosomal protein L26-2	Chloroplast (GO:0009507)	0.45
c15166.graph_c0	Psoralen synthase GN = CYP71AJ1	Chloroplast (GO:0009507)	0.44
c29431.graph_c0	2,3-bisphosphoglycerate-independent phosphoglycerate mutase	Chloroplast (GO:0009507)	0.44
c60534.graph_c0	DNA replication complex GINS protein psf1-like	Chloroplast (GO:0009507)	0.43
c869.graph_c1	Predicted protein GN = POPTRDRAFT_560048	Plastid (GO:0009536)	0.42
c43589.graph_c0	Deoxycytidylate deaminase	Chloroplast (GO:0009507)	0.41
c35959.graph_c0	Aquaporin PIP2-4	Chloroplast (GO:0009507)	0.41
c77513.graph_c0	Chromatin remodeling complex subunit (Fragment)	Chloroplast envelope (GO:0009941)	0.38
c18693.graph_c0	Dynamin-related protein 5A	Plastid (GO:0009536)	0.38
c16535.graph_c0	Elongation factor 2	Chloroplast (GO:0009507)	0.37
c33105.graph_c0	Pentatricopeptide repeat-containing protein At4g14820	Chloroplast (GO:0009507)	0.36
c14446.graph_c1	Transcription factor bHLH135, At1g74500	Plastid (GO:0009536)	0.35
c36899.graph_c2	alpha-xylosidase 1	Chloroplast (GO:0009507)	0.34
c19297.graph_c0	Histone H2B-like protein	Chloroplast (GO:0009507)	0.30
c42275.graph_c0	F-box/LRR-repeat protein 17	Chloroplast (GO:0009507)	0.29
c60647.graph_c0	Pentatricopeptide repeat-containing protein At4g21170	Plastid (GO:0009536)	0.28
c36899.graph_c0	alpha-xylosidase 1	Chloroplast (GO:0009507)	0.24

**Table 3:** DEGs related to important epigenetic modifications.

Gene name	Unigene	PL/SL
**DNA methylation and Histone H3-K9 methylation**		
WEB family protein At5g16730, chloroplastic (Precursor)	c37249.graph_c1	0.42
Serine/threonine-protein kinase TIO	c9096.graph_c0	0.22
Dynamin-related protein 5A	c18693.graph_c0	0.35
	c21060.graph_c0	0.44
	c38819.graph_c0	0.40
DNA polymerase alpha catalytic subunit	c28513.graph_c0	0.37
	c19609.graph_c1	0.31
	c74784.graph_c0	0.19
	c56991.graph_c0	0.25
DNA primase	c47274.graph_c0	0.27
Serine/threonine-protein kinase haspin-like	c19623.graph_c0	0.24
Structural maintenance of chromosomes protein 4	c40602.graph_c0	0.43
	c35582.graph_c0	0.23
125 kDa kinesin-related protein	c38826.graph_c0	0.28
	c38826.graph_c1	0.38
	c46154.graph_c0	0.42
DNA primase large subunit At1g67320	c40885.graph_c0	0.31
Uncharacterized protein	c17456.graph_c0	0.28
Myosin-6	c14817.graph_c0	0.30
Kinesin-like protein NACK2	c60343.graph_c0	0.27
Kinesin-1	c44221.graph_c0	0.36
Proline-rich receptor-like protein kinase PERK2	c51416.graph_c0	0.23
DNA replication licensing factor mcm2	c50980.graph_c0	0.31
Cell division control protein 45	c46105.graph_c2	0.31
MAR-binding filament-like protein 1	c15185.graph_c0	0.43
WD repeat and HMG-box DNA-binding protein 1-like	c48035.graph_c1	0.37
**DNA methylation**		
DNAMETHYLTRANSFERASE1 MET1	c42905.graph_c1	0.29
Meiotic nuclear division protein 1 homolog	c63574.graph_c0	0.21
**Histone H3-K9 methylation**		
Uncharacterized protein	c36435.graph_c0	0.45
Inactive leucine-rich repeat receptor-like protein kinase At1g66830	c46977.graph_c1	0.38
Microtubule-associated protein SPIRAL2-like	c25797.graph_c0	0.14
WEB family protein At4g27595, chloroplastic (Precursor)	c20350.graph_c1	0.24
Serine/threonine-protein kinase Aurora-1	c41902.graph_c0	0.38
Predicted protein	c869.graph_c1	0.38
**Histone H3-K27 methylation**		
Histone-lysine N-methyltransferase ASHR3	c61259.graph_c0	0.21
Histone-lysine N-methyltransferase ATXR6	c38866.graph_c0	0.24
**Histone lysine methylation**		
Structural maintenance of chromosomes protein 2-1	c1742.graph_c0	0.40
**Histone H3 acetylation**		
Methyl-CpG-binding domain-containing protein 9	c69530.graph_c0	0.43
