# Supplementary material for: Metabolic Flux Redirection and Transcriptomic Reprogramming in the Albino Tea Cultivar ‘Yu-Jin-Xiang’ with an Emphasis on Catechin Production
Source: Sci Rep. 2017 Mar 23;7:45062. doi: 10.1038/srep45062 (PMC5362904; doi:10.1038/srep45062)
Supplement: Supplementary Information [file srep45062-s1.pdf]

Metabolic Flux Redirection and Transcriptomic Reprogramming in the Albino Tea Cultivar 'Yu-Jin-Xiang' with an Emphasis on Catechin Production

Guo-Feng Liu[1], Zhuo-Xiao Han[1], Lin Feng[1], Li-Ping Gao[2], Ming-Jun Gao[3], Margaret Y Gruber[3,4], Zhao-Liang Zhang[1], Tao Xia[1], Xiao-Chun Wan[1], Shu Wei[1*]

[1]State Key Laboratory of Tea Plant Biology and Utilization, Anhui Agricultural University, 130 Changjiang Ave W., Hefei, Anhui, 230036, China

[2]College of Life Sciences, Anhui Agricultural University, Hefei, Anhui, 230036, China

[3]Agriculture and Agri-Food Canada, Saskatoon Research Centre, Saskatoon, SK, S7N 0X2, Canada

[4]Retired

*Corresponding author: weishu@ahau.edu.cn

Length Range	Contigs	Transcripts	Unigenes
0-300	3269895	43744	34342
300-500	29797	33827	21920
500-1000	17508	33102	12689
1000-2000	9787	39719	8153
2000+	4709	30769	5030
Total number	3331696	181161	82134
Total length	210648971	198943185	52097841
N50 length	71	1905	1061
Mean length	63.23	1098.16	634.30

Table S1 Assembly results of the library of *C. sinensis* cv. 'YJX'

Anno_Database	Annotated_Number	300<=length<1000	length>=1000
COG_Annotation	8459	2445	5146
GO_Annotation	23663	9318	9716
KEGG_Annotation	5864	2040	2869
Swissprot_Annotation	20807	8194	9020
TrEMBL_Annotation	33862	14326	12274
NR_Annotation	33780	14286	12276
All_Annotated	37753	16144	12460

Table S2 Annotation of the library of *C. sinensis* cv ‘YJX’

Gene number	Annotation	PL/SL
	**Pentatricopeptide repeat-containing protein**	
c29656.graph_c1	pentatricopeptide repeat-containing protein At3g13770	2.50
c31931.graph_c0	Pentatricopeptide repeat-containing protein At3g13150	2.37
c33502.graph_c0	Pentatricopeptide repeat-containing protein At5g16860	2.16
c57830.graph_c0	pentatricopeptide repeat-containing protein At1g10330	0.48
c32098.graph_c0	Pentatricopeptide repeat-containing protein At2g40240	0.40
c5120.graph_c0	Pentatricopeptide repeat-containing protein At5g64320	0.37
c38528.graph_c0	Pentatricopeptide repeat-containing protein At3g46790	0.28
	**UPS system**	
c27376.graph_c0	E3 ubiquitin-protein ligase Ring1	3.34
c33517.graph_c3	Ubiquitin-like modifier-activating enzyme atg7	2.78
c35227.graph_c0	E3 ubiquitin-protein ligase RHA2A	2.09
c40505.graph_c0	putative E3 ubiquitin-protein ligase RING1b	0.44
c42456.graph_c1	Ubiquitin-conjugating enzyme E2 19 GN=UBC19	0.31
	**Retrotransposon**	
c58415.graph_c0	copia-like retrotransposon	2.78
c3838.graph_c0	LINE-type retrotransposon LIb DNA	0.17
	**MEP pathway**	
c53960.graph_c0	Phy rapidly regulated 1	0.49

Table S3 Differentially expressed genes (*P*<0.05) related to pale green phenotype between pale leaves (PL) and shaded green leaves (SL) of ‘YJX’

Gene name	Primers	Purpose
VDE-1-F	CGACCTGATGAGACCGAATGCC	qPCR analysis for photo-protection
VDE-1-R	TCTGGGGCACACATTTCTTCCTT	qPCR analysis for photo-protection
VDE-2-F	TGGTTGAAGGGGAGATCACAAG	qPCR analysis for photo-protection
VDE-2-R	AGTGAAAGGTACGGTCATGGTC	qPCR analysis for photo-protection
ZEP-1-F	TAATGTTGAGTTGGGTCCTCGG	qPCR analysis for photo-protection
ZEP-1-R	AGGCACCACTCTCCATTAACAG	qPCR analysis for photo-protection
ZEP-2-F	CAACATTATCCTCAGCTTCGCG	qPCR analysis for photo-protection
ZEP-2-R	GCAGTTCTCTCTCCAAGGTCTC	qPCR analysis for photo-protection
APX3-F	TTGAGGATCCTAAGTTTCGTCCC	qPCR analysis for photo-protection
APX3-R	AGTAAACCCCAGCTCTGACAATT	qPCR analysis for photo-protection
GolS2-F	ACAGGAAGAGAACATGCAGAGAG	qPCR analysis for photo-protection
GolS2-R	ATCCATCCTCTGAGCTGTTCTTG	qPCR analysis for photo-protection
GSTF-F	CGATTTGGATAATGGAGAGCACAAA	qPCR analysis for photo-protection
GSTF-R	ATTATTGCCCTTGATTCGAATTGCT	qPCR analysis for photo-protection
E3 RING1-F	GAGTTAGCTATGTCGAGGGCTAG	qPCR analysis for chloroplast actvities
E3 RING1-R	CTTCTCCTTCTTCCTCCACCTTC	qPCR analysis for chloroplast actvities
ClpP5-F	AAGATCAACCAGGACACCGATC	qPCR analysis for chloroplast actvities
ClpP5-R	AGGCCGCAATAACTATGAGAGG	qPCR analysis for chloroplast actvities
LHCB7-F	GTACTTCACTGACACCACCACT	qPCR analysis for chloroplast actvities
LHCB7-R	TTGTTGGGGAAGATGGGATCAG	qPCR analysis for chloroplast actvities
TKRP125-F	AGCTTATCAAATGTGGCAAACTGAA	qPCR analysis for epigenetics
TKRP125-R	ACTTTTGTTTATCTCTCCTGCCTCT	qPCR analysis for epigenetics
MBD9-F	AGCAAGGAAGTTTACAAGGGAAATG	qPCR analysis for epigenetics
MBD9-R	ATCTTTCTTTTCAGGTTTGGGATGC	qPCR analysis for epigenetics
ANS-F	ATGCTTGTGGTCAACTTGAATGG	qPCR analysis for Flavonoid pathway
ANS-R	GAAGTTGCTTTGCGTACTCAACT	qPCR analysis for Flavonoid pathway
LAR1-F	AATTCACCATCAAAACCGTCGAC	qPCR analysis for Flavonoid pathway
LAR1-R	TCTTCTTCTCCCACAAAGATGCA	qPCR analysis for Flavonoid pathway
LAR2-F	GCTAGTACTCATCCACGCCATTA	qPCR analysis for Flavonoid pathway
LAR2-R	CCTCACCATTCGCTTTTCTTTGT	qPCR analysis for Flavonoid pathway
ANR1-F	CCCACTGAATTTGAACCAGCAAT	qPCR analysis for Flavonoid pathway
ANR1-R	TGTAGATGAGCCGCTTAACAGTT	qPCR analysis for Flavonoid pathway
ANR2-F	TCACTGTCATCCCAACTCTCATG	qPCR analysis for Flavonoid pathway
ANR2-R	CACAAACATCCTCTACGTGGGTA	qPCR analysis for Flavonoid pathway
F3H-F	AAGGTGGATTCATCGTTTCCAGT	qPCR analysis for Flavonoid pathway
F3H-R	GCAAGCCAAGTCCATCAATTTCT	qPCR analysis for Flavonoid pathway
CHS-F	AATAGCGAGCATAAGGTGGAGTT	qPCR analysis for Flavonoid pathway
CHS-R	CTCGCACACTAGAGGATTTTCTT	qPCR analysis for Flavonoid pathway
CHI-F	CTGTACACCCTTCGATCACTGAT	qPCR analysis for Flavonoid pathway
CHI-R	GATTTCCAAACCCCTCTCTCCTG	qPCR analysis for Flavonoid pathway
DFR-F	TCCTCTCTCTGTTTCTTCCTCCT	qPCR analysis for Flavonoid pathway
DFR-R	ATCTTCATCGAGATCTGCCTTCC	qPCR analysis for Flavonoid pathway
FLS-F	CTTCTTTGGGTGGTGATGAAATTGA	qPCR analysis for Flavonoid pathway
FLS-R	CCAATTACCGTCTTTCCAAACTTGA	qPCR analysis for Flavonoid pathway

To be continued

F3'H-F	TGGAACGTTAGAAGTGCCGTAA	qPCR analysis for Flavonoid pathway
F3'H-R	CCCGCTCTATCTACCCAACTTC	qPCR analysis for Flavonoid pathway
F3'5'H-F	ATGGGAATTTCAGCTGTCGAGT	qPCR analysis for Flavonoid pathway
F3'5'H-R	TGTAACACCAGTCCAAATGCCT	qPCR analysis for Flavonoid pathway
SAMDC-F	GGATGAAATTTTAACACCCGCTGA	qPCR analysis for Theanine pathway
SAMDC-R	CGAGTACACAAAGAGGCTTGATTC	qPCR analysis for Theanine pathway
GGT-1-F	AACAATTCCAAGGCCAAGTATGTG	qPCR analysis for Theanine pathway
GGT-1-R	TAGCGAGTTTTATTGAGGGTTGGA	qPCR analysis for Theanine pathway
GGT-2-F	CCATTCAAAAGGCCATTGTCATCT	qPCR analysis for Theanine pathway
GGT-2-R	TGTAGTCCCACCAATTATCATGCT	qPCR analysis for Theanine pathway
GOGAT-F	TGGTGTAGTTGATATTCCACCTGAG	qPCR analysis for Theanine pathway
GOGAT-R	GCTCTATCTTTTGTCTTTGAAGCCA	qPCR analysis for Theanine pathway
GS-F	ATGCCACACCAATTACAGTACAAAG	qPCR analysis for Theanine pathway
GS-R	TCATTTCCTTCTCCATAAGCACTGA	qPCR analysis for Theanine pathway
ALT-F	ACCCAGTTGCTTCATCAAGATAGTA	qPCR analysis for Theanine pathway
ALT-R	GCACTCTTGTAGGTTCATGCAATAT	qPCR analysis for Theanine pathway
GDH1-F	CAACCAACAGAAATTTTAAGCTGGC	qPCR analysis for Theanine pathway
GDH1-R	GAAGCCAAATTGCCATCATCTTTTG	qPCR analysis for Theanine pathway
NAD-GDH-F	TCATGCGCTTTTGTCAGAGTTTTAT	qPCR analysis for Theanine pathway
NAD-GDH-R	TAAAACTTCCCTGAACATGACTTGC	qPCR analysis for Theanine pathway
GDH2-F	GTGGGTCCAGAATATTCAAGGTTTC	qPCR analysis for Theanine pathway
GDH2-R	TCTGACACATGCTTTTGATGTTAC	qPCR analysis for Theanine pathway
TS-1-F	CCAAATTCCTTCCCAAATTCTCCAA	qPCR analysis for Theanine pathway
TS-1-R	TCCTTGACTACCTCCTTGTTGTTAG	qPCR analysis for Theanine pathway
TS-2-F	CAAAACTTGAAATCGTAGGCATCAC	qPCR analysis for Theanine pathway
TS-2-R	ACAACATAACATCGCCAATTGAAAG	qPCR analysis for Theanine pathway

Table S4 Primers used in this study for qPCR analysis

GO.ID	GO Term	Q-value
**Biological Process**		
GO:0006270	DNA-dependent DNA replication initiation	1.30E-06
GO:0006364	rRNA processing	1.70E-06
GO:0006098	pentose-phosphate shunt	2.60E-06
GO:0035304	regulation of protein dephosphorylation	2.90E-06
GO:0010207	photosystem II assembly	3.20E-06
GO:0010027	thylakoid membrane organization	8.70E-06
GO:0019288	isopentenyl diphosphate biosynthetic process, methylerythritol 4-phosphate pathway	1.00E-05
GO:0046686	response to cadmium ion	2.40E-05
GO:0009765	photosynthesis, light harvesting	2.60E-05
GO:0072594	establishment of protein localization to organelle	5.20E-05
GO:0000911	cytokinesis by cell plate formation	5.50E-05
GO:0050789	regulation of biological process	6.10E-05
GO:0006457	protein folding	7.60E-05
GO:0006269	DNA replication, synthesis of RNA primer	9.40E-05
GO:0008283	cell proliferation	0.00018
GO:0006605	protein targeting	0.0002
GO:0009451	RNA modification	0.00023
GO:0006275	regulation of DNA replication	0.00023
GO:0009773	photosynthetic electron transport in photosystem I	0.00024
GO:0009902	chloroplast relocation	0.00032
GO:0006306	DNA methylation	0.00032
GO:0009411	response to UV	0.00035
GO:0006996	organelle organization	0.00042
GO:0007076	mitotic chromosome condensation	0.00042
GO:0007067	mitosis	0.00064
GO:0010389	regulation of G2/M transition of mitotic cell cycle	0.0007
GO:0051567	histone H3-K9 methylation	0.00074
GO:0006511	ubiquitin-dependent protein catabolic process	0.00075
GO:0009639	response to red or far red light	0.00075
GO:0015995	chlorophyll biosynthetic process	0.00081
GO:0019344	cysteine biosynthetic process	0.00084
GO:0006486	protein glycosylation	0.00091
GO:0006635	fatty acid beta-oxidation	0.00099
GO:0032259	methylation	0.00099
GO:0048449	floral organ formation	0.00099
GO:0019252	starch biosynthetic process	0.00109
GO:0008361	regulation of cell size	0.00111
GO:0009108	coenzyme biosynthetic process	0.00127
GO:0048193	Golgi vesicle transport	0.00129
GO:0006950	response to stress	0.00142
GO:0016226	iron-sulfur cluster assembly	0.00146

To be continued

GO:0009966	regulation of signal transduction	0.00155
GO:0043161	proteasomal ubiquitin-dependent protein catabolic process	0.00166
GO:0007018	microtubule-based movement	0.00167
GO:0009704	de-etiolation	0.0017
GO:0017038	protein import	0.0017
GO:0006334	nucleosome assembly	0.00174
GO:0010023	proanthocyanidin biosynthetic process	0.00175
GO:0009651	response to salt stress	0.00197
GO:0009086	methionine biosynthetic process	0.00211
GO:0000394	RNA splicing, via endonucleolytic cleavage and ligation	0.00218
GO:0016567	protein ubiquitination	0.00233
GO:0000956	nuclear-transcribed mRNA catabolic process	0.00239
GO:0071461	cellular response to redox state	0.00249
GO:2000038	regulation of stomatal complex development	0.00256
GO:0016071	mRNA metabolic process	0.00277
GO:0006399	tRNA metabolic process	0.00295
GO:0051604	protein maturation	0.00303
GO:0009658	chloroplast organization	0.00303
GO:0015031	protein transport	0.00313
GO:0019684	photosynthesis, light reaction	0.00316
GO:0009926	auxin polar transport	0.00318
GO:0009793	embryo development ending in seed dormancy	0.00332
GO:0008033	tRNA processing	0.00353
GO:0009753	response to jasmonic acid stimulus	0.00355
GO:0065003	macromolecular complex assembly	0.00363
GO:0051187	cofactor catabolic process	0.00415
GO:0016117	carotenoid biosynthetic process	0.00418
GO:0044265	cellular macromolecule catabolic process	0.0043
GO:0009165	nucleotide biosynthetic process	0.00439
GO:0051788	response to misfolded protein	0.00445
GO:0043248	proteasome assembly	0.00445
GO:0048585	negative regulation of response to stimulus	0.00456
GO:0015916	fatty-acyl-CoA transport	0.00456
GO:0006626	protein targeting to mitochondrion	0.00456
GO:0090304	nucleic acid metabolic process	0.00469
GO:0009069	serine family amino acid metabolic process	0.00516
GO:0048513	organ development	0.00517
GO:0007129	synapsis	0.00521
GO:0010048	vernalization response	0.00525
GO:0071841	cellular component organization or biogenesis	0.00619
GO:0018130	heterocycle biosynthetic process	0.00623
GO:0042793	transcription from plastid promoter	0.0063
GO:0018193	peptidyl-amino acid modification	0.00642
GO:0009073	aromatic amino acid family biosynthetic process	0.00652
GO:0009965	leaf morphogenesis	0.00672

To be continued

GO:0007031	peroxisome organization	0.0068
GO:0006273	lagging strand elongation	0.00733
GO:0080005	photosystem stoichiometry adjustment	0.0074
GO:0009627	systemic acquired resistance	0.00742
GO:0007017	microtubule-based process	0.00751
GO:0009628	response to abiotic stimulus	0.0076
GO:0007349	cellularization	0.00761
GO:0006487	protein N-linked glycosylation	0.00775
GO:0071822	protein complex subunit organization	0.00781
GO:0006446	regulation of translational initiation	0.00795
GO:0019243	methylglyoxal catabolic process to D-lactate via S-lactoyl-glutathione	0.00796
GO:0010388	cullin deneddylation	0.008
GO:0008380	RNA splicing	0.00809
GO:0045893	positive regulation of transcription, DNA-templated	0.00811
GO:0006470	protein dephosphorylation	0.00837
GO:0006661	phosphatidylinositol biosynthetic process	0.0084
GO:0005996	monosaccharide metabolic process	0.0085
GO:0010228	vegetative to reproductive phase transition of meristem	0.00855
GO:0019761	glucosinolate biosynthetic process	0.00869
GO:0009617	response to bacterium	0.00894
GO:0051716	cellular response to stimulus	0.00896
GO:0000302	response to reactive oxygen species	0.00916
GO:0070271	protein complex biogenesis	0.00933
GO:0006766	vitamin metabolic process	0.0094
GO:0015804	neutral amino acid transport	0.00943
GO:0034660	ncRNA metabolic process	0.00946
GO:0010646	regulation of cell communication	0.00947
GO:0015994	chlorophyll metabolic process	0.00954
**Cellular Component**		
GO:0009507	chloroplast	5.30E-10
GO:0009570	chloroplast stroma	2.00E-08
GO:0009941	chloroplast envelope	3.60E-07
GO:0009522	photosystem I	1.00E-06
GO:0005829	cytosol	4.90E-05
GO:0009535	chloroplast thylakoid membrane	8.30E-05
GO:0010287	plastoglobule	0.00022
GO:0009579	thylakoid	0.00037
GO:0000796	condensin complex	0.0004
GO:0005871	kinesin complex	0.00079
GO:0009523	photosystem II	0.00094
GO:0031090	organelle membrane	0.0011
GO:0005874	microtubule	0.00159
GO:0048046	apoplast	0.00191
GO:0000786	nucleosome	0.0027

To be continued

GO:0005732	small nucleolar ribonucleoprotein complex	0.00313
GO:0046861	glyoxysomal membrane	0.00423
GO:0005802	trans-Golgi network	0.00423
GO:0030076	light-harvesting complex	0.00501
GO:0009706	chloroplast inner membrane	0.00553
GO:0044429	mitochondrial part	0.00559
GO:0044464	cell part	0.00594
GO:0010008	endosome membrane	0.00746
GO:0005794	Golgi apparatus	0.00857
GO:0031984	organelle subcompartment	0.00883
GO:0009508	plastid chromosome	0.00889
GO:0005634	nucleus	0.00947
**Molecular Function**		
GO:0005515	protein binding	4.40E-09
GO:0016853	isomerase activity	0.00013
GO:0016168	chlorophyll binding	0.00022
GO:0005200	structural constituent of cytoskeleton	0.00022
GO:0008017	microtubule binding	0.00079
GO:0005525	GTP binding	0.00086
GO:0000156	two-component response regulator activity	0.00096
GO:0003896	DNA primase activity	0.00125
GO:0008184	glycogen phosphorylase activity	0.00136
GO:0051082	unfolded protein binding	0.00163
GO:0016874	ligase activity	0.00175
GO:0046983	protein dimerization activity	0.00178
GO:0003777	microtubule motor activity	0.00227
GO:0005083	small GTPase regulator activity	0.00229
GO:0004857	enzyme inhibitor activity	0.00244
GO:0009044	xylan 1,4-beta-xylosidase activity	0.00275
GO:0050589	leucocyanidin oxygenase activity	0.00368
GO:0015450	P-P-bond-hydrolysis-driven protein transmembrane transporter activity	0.00371
GO:0016835	carbon-oxygen lyase activity	0.00389
GO:0031418	L-ascorbic acid binding	0.00397
GO:0004842	ubiquitin-protein ligase activity	0.00483
GO:0016841	ammonia-lyase activity	0.00484
GO:0016667	oxidoreductase activity, acting on a sulfur group of donors	0.00543
GO:0016682	oxidoreductase activity, acting on diphenols and related substances as donors, oxygen as acceptor	0.00568
GO:0052689	carboxylic ester hydrolase activity	0.00569
GO:0031072	heat shock protein binding	0.00607
GO:0035173	histone kinase activity	0.00674
GO:0008168	methyltransferase activity	0.0068
GO:0048037	cofactor binding	0.00926

Table S5 Significantly changed Gene Ontology (GO) terms in pale leaves compared to shaded green leaves

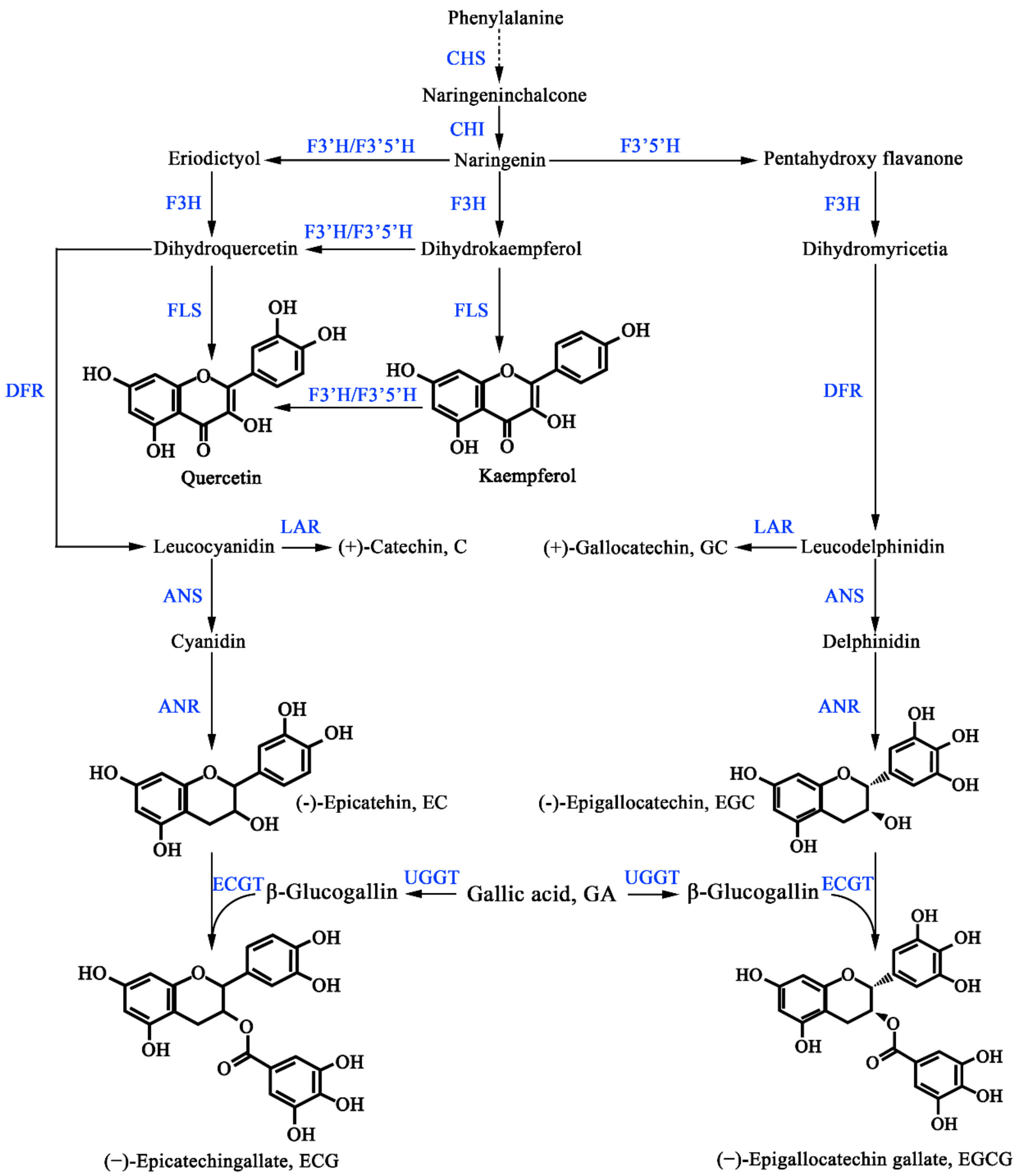

Figure S1. Schematic diagram of flavonoid pathway.
ANR: anthocyanidin reductase; ANS: anthocyanidin synthase; CHI, chalcone isomerase; CHS, chalcone synthase; DFR, dihydroflavonol 4-reductase; F3H, flavanone 3-hydroxylase; F3′H, flavonoid 3′-hydroxylase; F3′5′H, flavonoid 3′,5′-hydroxylase; FLS, flavonol synthase; LAR, leucoanthocyanidin reductase.

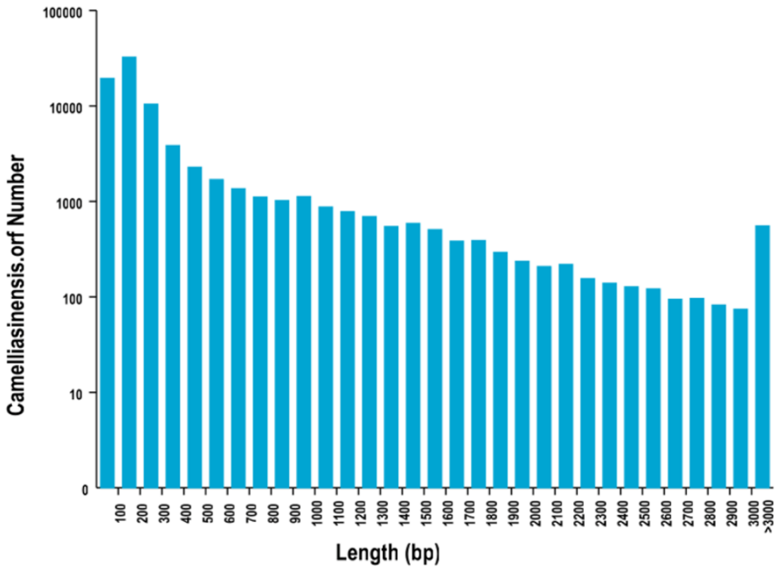

Figure S2. Detailed information of ORFs distribution genome wide.

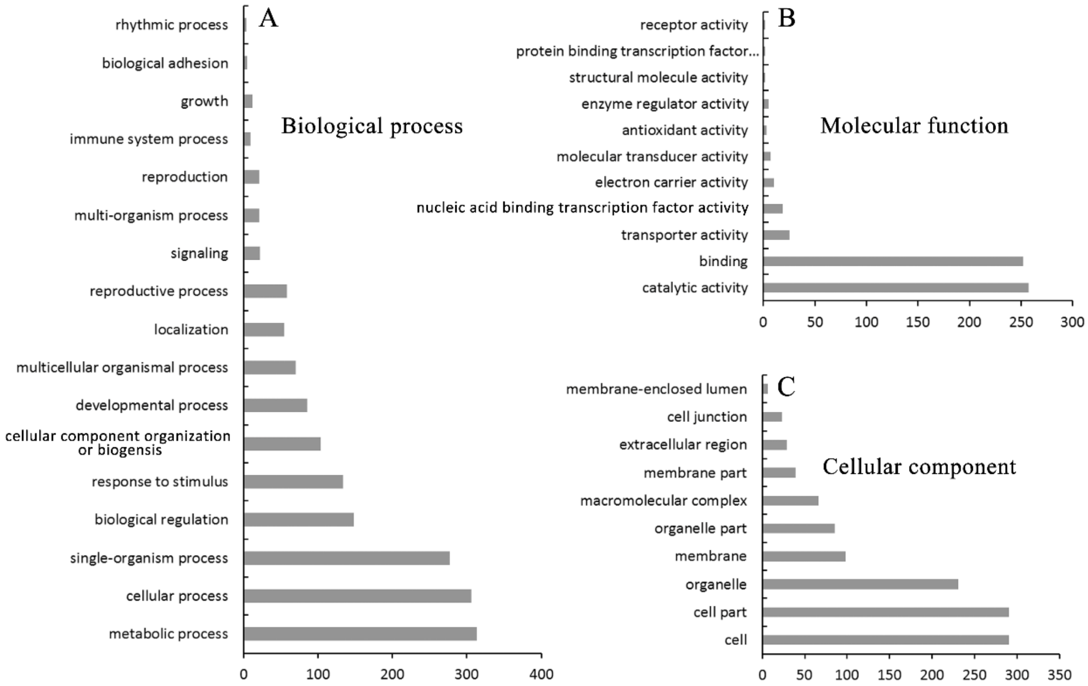

Figure S3. Overpresented gene ontology biological processes (A), molecular functions (B) and cellular components (C) out of 355, 102 and 64 for the three categories respectively due to the differentially expressed genes between the non-shaded pale leaves and shaded leaves on 'YJX'.
